# MSH6 germline mutations leading to Lynch syndrome-associated cholangiocarcinoma: a case report

**DOI:** 10.3389/fonc.2024.1414665

**Published:** 2024-08-05

**Authors:** Zheng Zhang, Subo Ma, Shixing Li, Zhengfu Chen, Runda Song, Zhanpeng Wang

**Affiliations:** Department of hepatobiliary and pancreatic surgery, China-Japan Union Hospital, Jilin University, Changchun, China

**Keywords:** Lynch Syndrome, cholangiocarcinoma, mismatch repair genes, immunohistochemistry, MSH6 gene

## Abstract

Lynch syndrome, a hereditary cancer susceptibility syndrome, arises from pathogenic mutations in mismatch repair genes. This syndrome is strongly linked to colorectal and endometrial cancers, as well as an elevated risk for other cancers such as gastric, ovarian, renal pelvis/ureter, and prostate. Notably, Lynch syndrome is rarely associated with cholangiocarcinoma (CCA). In this case study, we present a unique instance of Lynch syndrome-related CCA resulting from a singular MSH6 mutation. Notably, our findings reveal discrepancies between immunohistochemistry (IHC) and microsatellite stability results compared to genetic testing outcomes. This discrepancy underscores the limitations of solely relying on IHC analysis and microsatellite stability testing for the detection of hereditary tumors, emphasizing the crucial role of genetic testing in such cases. This insight enhances our comprehension of the mechanisms involved in cancer development and underscores the significance of thorough analysis integrating immunohistochemistry and genetic testing for diagnosing Lynch syndrome-related cancers.

## Background

Cholangiocarcinoma (CCA) is a malignant tumor that arises from the epithelial cells of the bile duct. The exact pathogenesis of this cancer remains unclear. While the majority of biliary tract cancers occur sporadically, it is believed that approximately 10%-15% of CCAs may have a genetic component (1). At least four genetic conditions increase the risk of CCA, including Lynch syndrome (2). Lynch syndrome is a rare hereditary cancer susceptibility syndrome caused by pathogenic germline mutations in mismatch repair (MMR) genes. The majority of Lynch syndrome cases are associated with microsatellite instability (MSI) (3, 4). The vast majority of MMR genomic changes will cause the loss of MMR protein expression, usually manifested by the corresponding loss of immunohistochemical (IHC) staining. In some cases, there may be inconsistencies between MMR mutations and IHC results. Previous studies (5) have suggested that these discrepancies could be attributed to somatic missense mutations leading to the production of non-functional antigenic proteins. This could result in false negative IHC results.

We report a unique CCA associated with Lynch syndrome, combine it with comprehensive genomic analysis to explore its unique pathological phenotype and molecular mechanism, and provide new insights for the clinical diagnosis of Lynch syndrome.

## Case presentation

A 72-year-old female presented with jaundiced skin and underwent bile duct puncture and drainage at a local hospital. Seeking further treatment, she was referred to the China-Japan Union Hospital of Jilin University. In addition to her primary symptoms, the patient also presented with jaundice of the sclera and general itching. Her medical and family history showed no significant abnormalities. Laboratory tests revealed elevated levels of carbohydrate antigen 19-9 (CA19-9), carbohydrate antigen 242 (CA24-2), total bilirubin (T-bil), and direct bilirubin (D-bil), while total protein (TP) and albumin (ALB) levels were slightly below normal. Apart from these findings, the general examination did not reveal any significant abnormalities. Some serological tests of this patient are shown in **Table 1**.

**Table 1:** Preoperative related serological index results.

Factor	Normal range	Pre-operation
CA19-9 (U/ml)	**≤30**	641.10
CA24-2 (U/ml)	**<20**	80.98
ALT (IU/L)	5-40	37.45
AST (IU/L)	8-40	47.19
T-bil (μmol/L)	5-21	233.11
D-bil (μmol/L)	0-3.4	115.16
TP (g/L)	62-38	61.91
ALB (g/L)	35-52	33.65

CA19-9, carbohydrate antigen 199; CA24-2, carbohydrate antigen 242; ALT, alanine aminotransferase; AST, aspartate aminotransferase; T-bil, total bilirubin; D-bil, direct bilirubin; TP, total protein; ALB, albumin.

**Figure 1:**
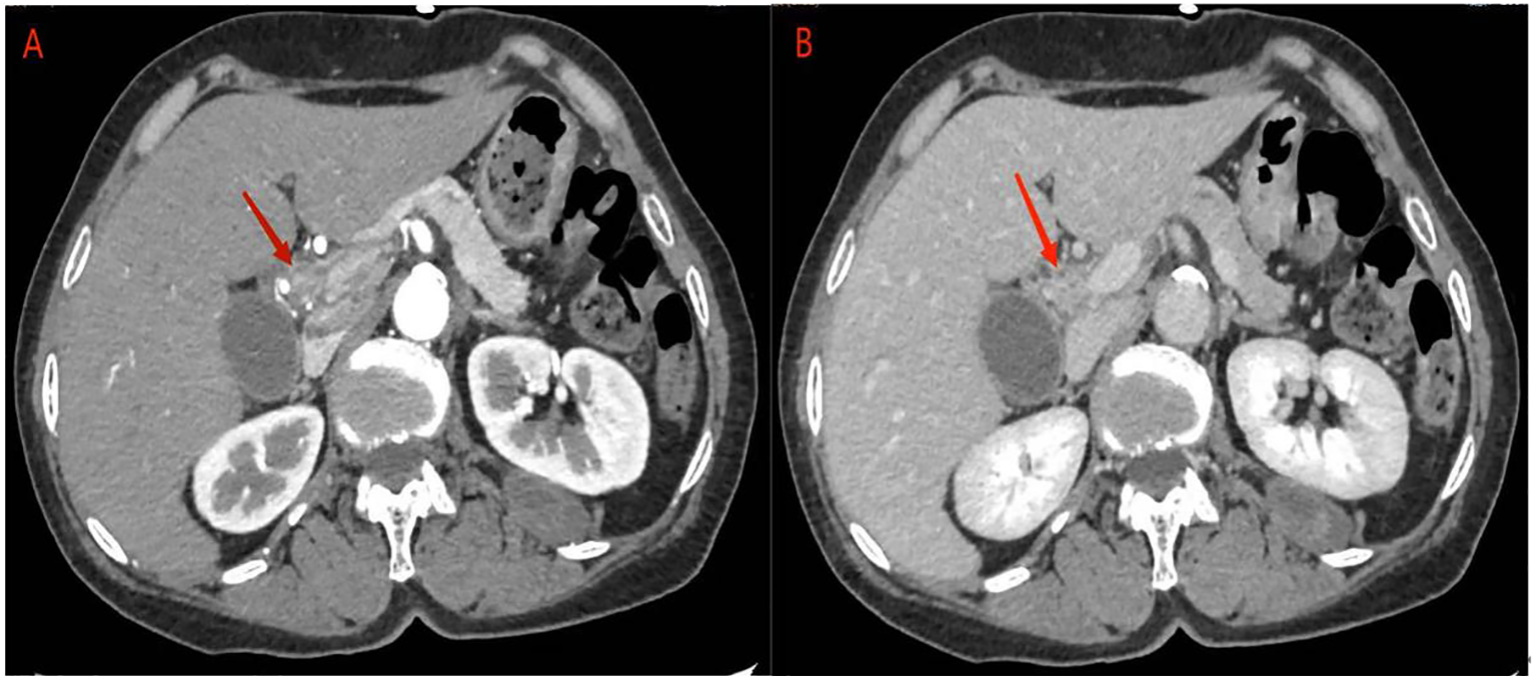
abdominal contrast-enhanced CT: **(A)** the contrast-enhanced scan of the tumor showed significant enhancement, corresponding to narrowing of the bile duct lumen. **(B)** In the delayed phase, tumor enhancement may be reduced.

Abdominal ultrasonography revealed a hypoechoic mass in the lower section of the common bile duct. Subsequent abdominal contrast-enhanced CT demonstrated uneven thickening of the duct wall above the pancreas, significant enhancement in the contrast-enhanced scan, narrow lumen, along with an enlarged gallbladder and upper bile duct (**Figure 1**). These findings collectively indicate CCA.

**Figure 2:**
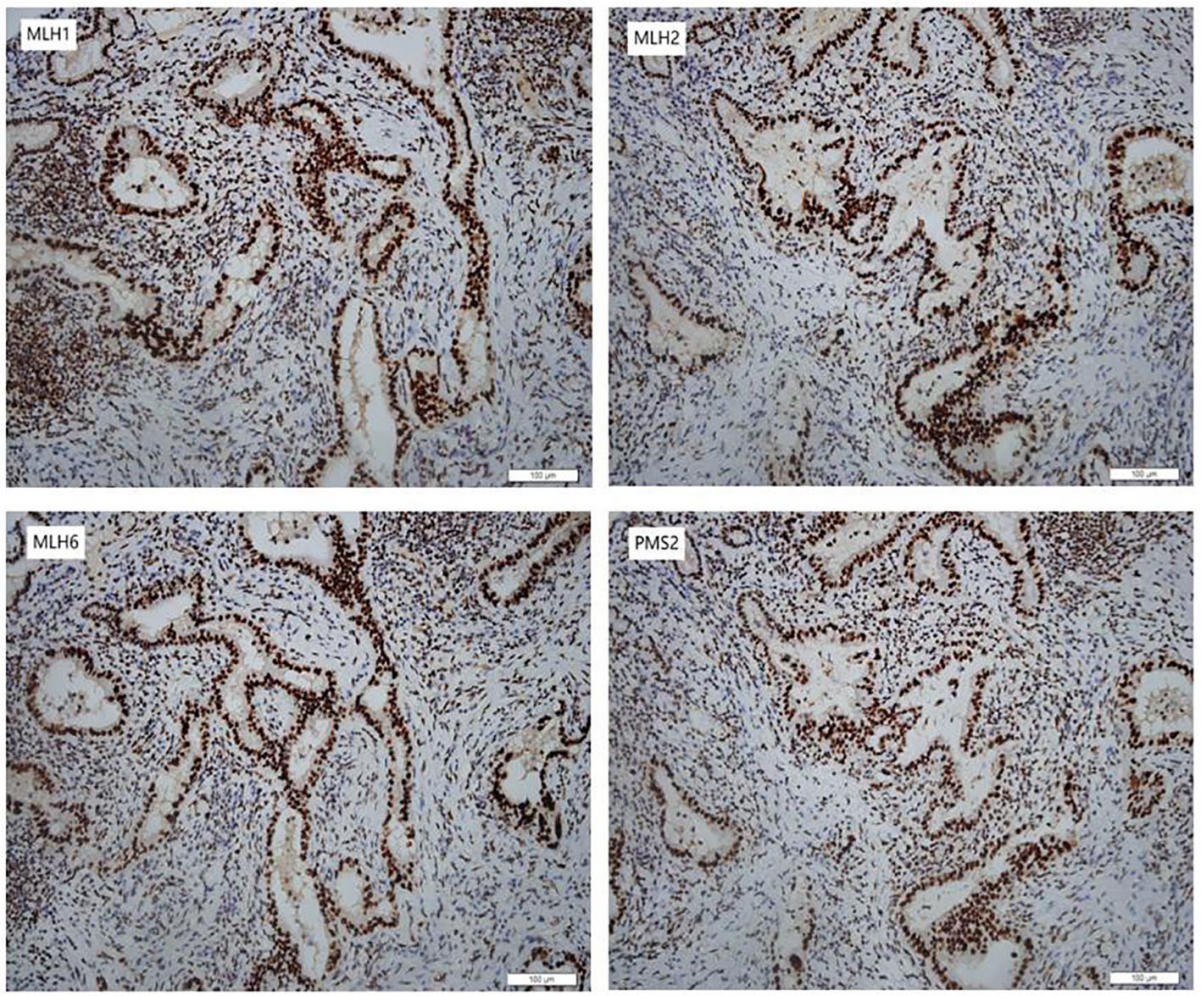
Immunohistochemical staining of the CCA. The CCA was positive for MLH1 (MutL Homolog 1), MSH2 (MutS Homolog 2), MSH6 (MutS Homolog 6) and PMS2 (PMS1 Homolog 2) (200×).

In January 2024, she underwent robotic-assisted pancreaticoduodenectomy and abdominal drainage. Postoperative pathological examination revealed a 2.0*1.2*0.5cm CCA. The tumor had invaded the full thickness of the bile duct wall, with cancer tissue showing necrosis and interstitial fibrosis. TNM stage is T_1_N_1_M_0_. Surgical margins were negative, and IHC indicated for MLH1(+), MSH2(+), MSH6(+) and PMS2(+) in the tumor (**Figure 2**).

Genomic analysis revealed a Lynch syndrome-related MSH6 germline pathogenic mutation in the patient, specifically a base substitution (c.3964G>T) within the MSH6 gene. However, the tumor was classified as microsatellite stable (MSS) (**Table 2**).

**Table 2:** The patient’s genetic test results.

Gene	Specific variations	Clinical significance	Familial hereditary tumor syndrome	Zygote type	Inheritance mode
MSH6	c.3964G>T (p.E1322*)	Pathogenic variant	Lynch syndrome	Heterozygote	Autosomal dominant

The patient recovered well post-surgery without any complications and was discharged from the hospital on the 19th day post-operation. Currently, the patient is undergoing outpatient follow-up with no signs of recurrence.

## Discussion

There are limited case reports on Lynch syndrome-associated CCA. Research suggests that Lynch syndrome may elevate the risk of various malignancies, with gastrointestinal, endometrial, ovarian, and urinary tract cancers being the most common. Lynch syndrome-related biliary system tumors make up only 1.4% to 4% of cases (6). MMR genes associated with Lynch syndrome include MutL Homolog 1 (MLH1), MutS Homolog 2 (MSH2), MutS Homolog 6 (MSH6), and PMS1 Homolog 2 (PMS2). Under typical conditions, MLH1 and MSH2 have the ability to create stable heterodimers with other proteins, whereas PMS2 and MSH6 can only interact with MLH1 and MSH2, respectively. As a result, in IHC analysis of MMR protein expression, the common pattern observed is the presence of MLH1 and PMS2. It is important to note that deletion of MSH2 and MSH6 usually occurs simultaneously, while PMS2 or MSH6 deletions can occur independently (7).

The case we report is the first case of Lynch syndrome-related CCA caused by a single MSH6 germline mutation. Cloyd et al. (8) examined 11 cases of biliary-related malignant tumors associated with Lynch syndrome, with the exception of 1 case involving the ampulla of Vater. The findings revealed that all cases were linked to defects in MSH1 and MSH2, while no correlation was observed between CCA and MSH6 deficiency. A previous investigation in a family registry also did not identify MSH6-related CCA (9). Although some studies have reported cases of CCA with concurrent deletions of MSH6 and MSH2, these studies did not conclusively identify specific germline mutations (10, 11).

The case involved a 72-year-old individual with a tumor located at the lower end of the common bile duct, diagnosed as bile duct adenocarcinoma. Comprehensive genomic analysis identified a base substitution in exon 9 of the MSH6 gene, leading to a missense mutation in the encoded amino acid (c.3964G>T, p.E1322*). These germline mutations are classified as pathogenic variants. Despite this, IHC results indicated the presence of MSH6 protein expression, and microsatellite stability testing confirmed a microsatellite stable (MSS) status.

One intriguing aspect of this case is that despite the pathogenic mutation in MMR resulting in tumor formation, the IHC test revealed proficient mismatch repair function (pMMR). This discrepancy could be attributed to the mutation causing a loss of protein function, while preserving its antigenic epitope. Therefore, IHC revealed expression of all four proteins. Previous research suggested (12) that Lynch syndrome, may result from a missense mutation affecting protein function, however, this mutation did not lead to intracellular protein degradation.

In addition, the patient was MSS. While this condition is uncommon, it has been documented in cases of MSH6 mutations (13). Despite the fact that the MSH6 gene plays a role in DNA, MSS may manifest in certain tumors with germline mutations in MSH6 (14, 15). The current understanding suggests that the situation may be attributed to two main factors: 1, MSH6 is primarily responsible for correcting base-base mismatches, leading to a lack of MSI in mutant cells due to the accumulation of base substitution mutations (16); 2, There is partial functional redundancy between MSH6 and MSH3 proteins, allowing MSH3 to partially compensate for MSH6 defects in correcting DNA mismatches (17, 18).

In addition, the patient had no family history of hereditary cancer associated with Lynch syndrome, which challenged our diagnosis. Lynch syndrome is an autosomal dominant genetic disease known for its familial aggregation characteristics. In the past, clinical diagnostic criteria for Lynch syndrome, heavily relied on family history (19, 20). However, numerous reports and extensive research have indicated that family history may exhibit lower sensitivity and specificity in the diagnosis of Lynch syndrome. Studies have shown (21) that 40% of Lynch syndrome, patients do not have family histories that align with genetic screening criteria. As a result, some studies recommend disregarding family history altogether. In 2009, Lynch et al. (22) established germline mutations in MMR as defining characteristics of Lynch syndrome.

There is currently no clear evidence-based evidence and guideline recommendations for the treatment of cholangiocarcinoma associated with Lynch syndrome caused by MSH6 germline mutations. Surgical resection remains the primary treatment option for resectable MMR-related CCA. In this patient’s case, along with the presence of MSH6 pathogenic variants, there are also alterations in homology-dependent recombination repair (HRR) pathway-related genes like AT-rich interactive domain (ARID1A) and TP53. These findings suggest that the patient could potentially benefit from immune checkpoint inhibitor therapy and poly ADP-ribosepolymerase (PARP) enzyme inhibitor therapy. Genomic alterations in DNA damage repair pathways are emerging as promising targets for treating various tumors (23). Initially utilized in patients with BRCA gene mutations, PARP inhibitors not only impede DNA repair and trigger apoptosis in BRCA mutant cells, but also impact HRR-related pathways (24). Furthermore, research indicates that defects in the HRR pathway within tumors can potentially boost immune recognition and targeting by promoting the generation of neoantigens, thereby augmenting immune-based therapies (25). The pembrolizumab monoclonal antibody has been approved for treating pan-solid tumors with deficient mismatch repair (dMMR) (26). Future treatments should focus on comprehensive genomic analysis and pathological characterization. This will not only assess the effectiveness and safety of anti-PD-1 or anti-PD-L1 therapy in these patients but also allow for combination therapy targeting multiple pathways to improve survival outcomes.

## Summary and conclusion

The incidental discovery of Lynch syndrome carries significant clinical implications for both patients and their families. This case serves as a reminder that hereditary tumors may not be fully detected solely through IHC analysis and microsatellite stability testing of MMR genes, highlighting the crucial role of genetic testing. MMR gene germline mutation detection is the gold standard diagnostic method for Lynch syndrome, and cancer type and family history should be ignored.

## Data availability statement

The original contributions presented in the study are included in the article/supplementary material. Further inquiries can be directed to the corresponding author/s.

## Ethics statement

The studies involving humans were approved byEthics Committee of China-Japan Union Hospital of Jilin University. The studies were conducted in accordance with the local legislation and institutional requirements. The participants provided their written informed consent to participate in this study. Written informed consent was obtained from the individual(s) for the publication of any potentially identifiable images or data included in this article.

## Author contributions

ZZ: Conceptualization, Data curation, Formal analysis, Funding acquisition, Investigation, Methodology, Project administration, Resources, Software, Supervision, Validation, Visualization, Writing – original draft, Writing – review & editing. SM: Conceptualization, Data curation, Investigation, Resources, Writing – review & editing. SL: Data curation, Writing – review & editing. ZC: Data curation, Writing – review & editing. RS: Data curation, Writing – review & editing. ZW: Data curation, Supervision, Validation, Writing – original draft, Writing – review & editing.
